# Mouse model of subtalar post-traumatic osteoarthritis caused by subtalar joint instability

**DOI:** 10.1186/s13018-022-03435-4

**Published:** 2022-12-12

**Authors:** Shuo Wang, Peixin Liu, Kaiwen Chen, Hongtao Zhang, Jia Yu

**Affiliations:** 1 Department of Orthopedics, The First Affiliated Hospital of Soochow University, 899 Pinghai Road, Suzhou, 215006 Jiangsu People’s Republic of China; 2 Orthopaedic Institute, Medical College, Soochow University, 178 Ganjiangdong Rd, Suzhou, 215006 Jiangsu People’s Republic of China; 3 Department of Orthopedics, Suzhou Xiangcheng People’s Hospital, 1060 Huayuan Road, Suzhou, 215131 Jiangsu People’s Republic of China

**Keywords:** Ankle sprain, Ankle instability, Subtalar instability, Post-traumatic osteoarthritis, Calcaneofibular ligament

## Abstract

**Background:**

Common ankle sprains are often accompanied by injury to the subtalar joint, which eventually leads to subtalar joint instability. Because the clinical manifestations for subtalar joint instability are similar to ankle joint injuries, these are often overlooked. This study aimed to establish an animal model of subtalar joint instability to study post-traumatic osteoarthritis of the subtalar joint caused by long-term subtalar joint instability and to provide a reference for future clinical research on chronic subtalar joint instability.

**Methods:**

In all, 24 C57BL/6 male mice were randomly divided into three groups: Sham, cervical ligament (CL) transection and CL + calcaneofibular ligament (CFL) transection groups. One week after surgical operation, all mice were trained to run in the mouse rotation fatigue machine every day. During this period, a balance beam test was used to evaluate the motor level and coordination ability of the mice before the operation and three days, one week, four weeks, eight weeks, and twelve weeks after operation. Further, post-traumatic osteoarthritis of the subtalar joint was quantified via micro-CT and histological staining.

**Results:**

The mice in the partial ligament transection group took significantly longer than those in the Sham group to pass through the balance beam and showed an increased number of hindfoot slips. Micro-CT analysis showed that the subtalar bone volume fraction in the CL + CFL transection group and CL transection group was 5.8% and 2.8% higher than that in the Sham group, respectively. Histological staining showed obvious signs of post-traumatic osteoarthritis (PTOA) in the subtalar joint of the ligament transection group.

**Conclusions:**

The transection of CL and CL + CFL can cause instability of the subtalar joint in mice, resulting in a decrease in motor coordination, and long-term instability of the subtalar joint in mice can cause PTOA of the subtalar joint, which is manifested as destruction and loss of articular cartilage.

## Introduction

Ankle sprain (AS) is one of the most common athletic injuries occurring during sports activities. Among all AS cases, approximately 25% patients present with combined injuries of the lateral ankle and the subtalar joint [1–3]. However, because of similar mechanisms of injury and clinical manifestations (both may develop symptoms such as ecchymosis, hematoma, or tenderness on the lateral foot), the involvement of subtalar joint is often neglected [1, 4, 5]. Undetected subtalar joint injuries may result in chronic subtalar instability (CSI) and further cause mechanical and functional instability in the hindfoot, ultimately leading to pain, dysfunction, joint deformity, and PTOA [2, 4, 6, 7]. The consequences not only increase the financial burden of families but also seriously affect patient quality of life. Therefore, to prevent irreversible traumatic osteoarthritis, early diagnosis and treatment of subtalar instability remain to be an urgent issue to be solved.

At present, there are several reports on chronic ankle instability (CAI), and experts have reached consensus on its diagnosis and treatment [8]. However, the literature on chronic subtalar instability is limited, and the diagnosis and treatment thereof are challenging. Subtalar instability has been observed to be related to around-the-talus ligamentous injuries, involving either the intrinsic ligaments, including interosseous talocalcaneal ligament (ITCL) and cervical ligament (CL), or the extrinsic ligaments, including calcaneofibular ligament (CFL) and deltoid ligament (DL). CFL, as the only ligament that connects the tibiotalar and subtalar joints, is deemed to play a crucial role in the stability of the two joints [9, 10]. Kamada et al. [11] reported that chronic subtalar instability may be attributed to CFL rupture. Other researchers believe that one of the intrinsic ligaments of subtalar joint, i.e., CL, contributes more to subtalar stability [12, 13]. A study by Martin [14] showed that in the event of complete ligament rupture of the CFL, the strain of CL plays an important role in maintaining the stability of the subtalar joint.

To date, the exact mechanism of injuries causing subtalar instability remains unknown, and the impact of ligaments around the subtalar joint on subtalar stability remains a controversial topic. Thus, the anatomical structure of the subtalar joint and the distribution of ligaments around the subtalar joint in C57BL/6 mice are similar to those in humans [15]. They are viable experimental animals for constructing animal models. Chang et al. [15] successfully developed three different ankle osteoarthritis models using mice. Liu et al. [16] used the instability of mouse ankle joint–subtalar joint complex to successfully establish a PTOA model. Therefore, mouse models have been widely used to study various joint diseases. Inspired by mouse models with ankle instability and ankle–subtalar joint complex instability, as well as our team's existing techniques and previous research experience [16, 17]. We believed that this problem of establishing a mouse model of PTOA caused by subtalar joint instability may be investigated via a similar approach.

We hypothesized that transecting the ligaments around the subtalar joint of the mouse could lead to chronic subtalar joint instability accompanied by subtalar joint osteoarthritis. To test this hypothesis, we designed two mouse models of chronic subtalar joint instability accompanied by subtalar joint osteoarthritis induced by ligament disruption (transection of CL alone or transection of CL and CFL). In this study, the balancing results were used to evaluate the behavioral stability of mice, and the results of specimens subject to micro-CT, safranin O-Fast green staining, hematoxylin and eosin (H&E) staining, and immunohistochemical staining were employed to evaluate inflammation of the subtalar joint. The successful establishment of the mouse model of chronic subtalar joint instability accompanied by subtalar joint osteoarthritis not only provides a new understanding for the research of foot diseases, but also provides a reference for clinical research on the mechanism and treatment of injury of chronic subtalar joint instability.

## Methods

### Animals

In all, 24 male C57BL/6 mice [age, 7 weeks; ranging from 18.1 to 24.6 g (mean body weight: 20.3 g)] purchased from JOINN Laboratories (Suzhou), Inc. (License No. SCXK (Su) 2018-0006, Suzhou, China) were used in the present experiment. All mice were randomly distributed into six plastic cages, with an average of four mice per cage. The mice were housed in a specific pathogen-free (SPF) environment with a light/dark cycle of 12 h, and constant temperature and humidity of 18–22 °C and 40–70%, respectively. The mice were free to move around in the cage and were provided with adequate food and water. The beddings in the cages were changed regularly, and the health of the mice was carefully observed. In the first week, the mice were allowed to adapt to the new feeding environment and were trained on the balance beam.

### Surgical procedures

**Fig. 1:**
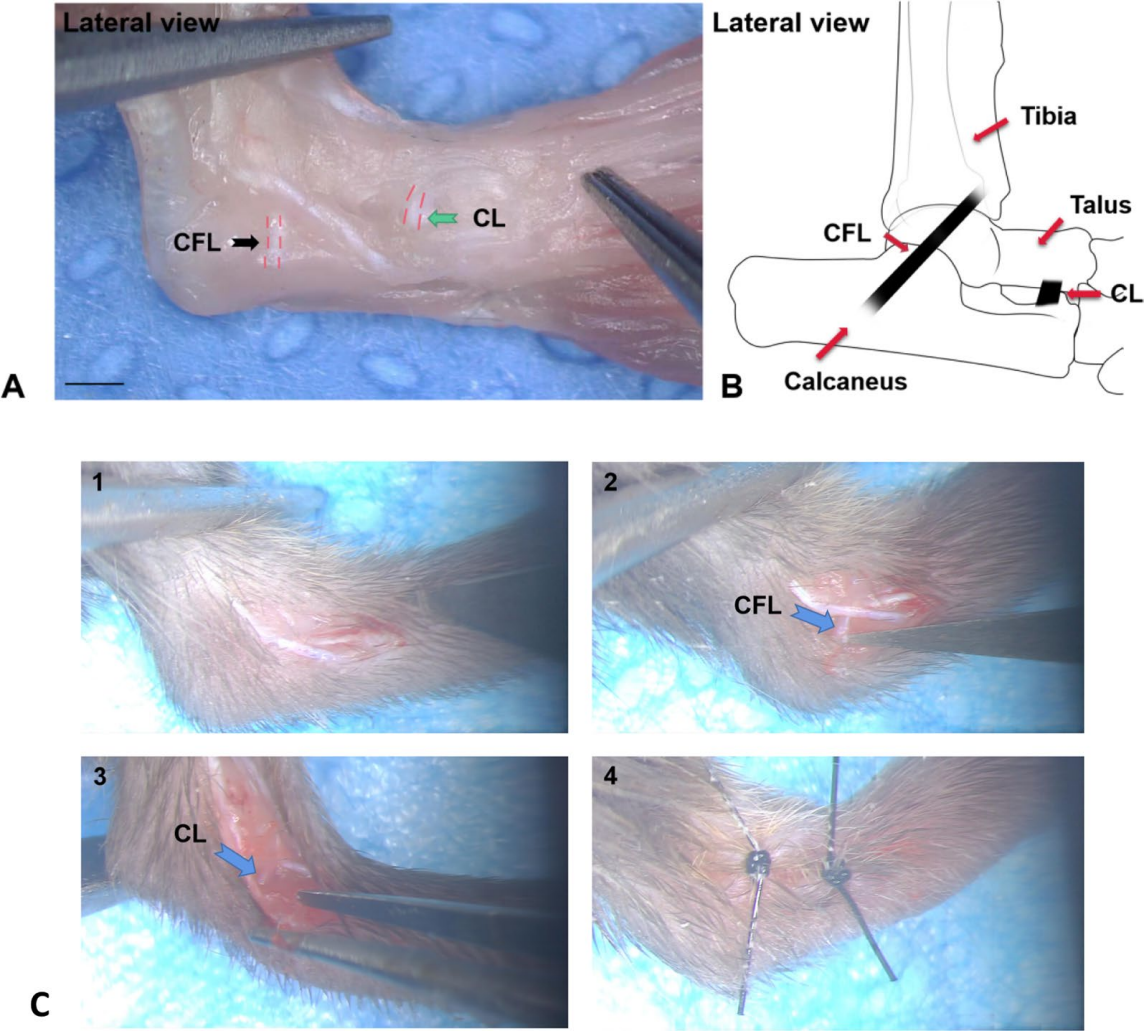
Anatomy and location of ankle ligament in mice. **A** Location of the calcaneofibular ligament and the cervical ligament, lateral view. **B** Schematic diagram of the calcaneofibular ligament and the cervical ligament. **C** Main surgical procedures from making a incision to suturing wound under the microscopical view. (1—make a 4 mm oblique downward longitudinal incision on the skin of the lateral side of right ankle joint. 2—Expose calcaneofibular ligament (CFL) and transect the CFL. 3—Expose cervical ligament (CL) and transect the CL. 4—Suture with two stitches using 5-0 surgical nylon thread.) Scale bar = 1 mm

At 8 weeks of age, the six cages of mice were randomly divided into three groups, with two cages (eight mice) per group. The mice were weighed before surgery, and anesthetized via an intraperitoneal injection of 4% chloral hydrate at a dose of 0.01 ml/g. A shaver was then used to clean the hair on the right hind limbs of the anesthetized mice, and their right hind limbs were disinfected in strict accordance with the principles of surgical disinfection. Following this, the disinfected mice were placed on a microsurgical operating table, and the anatomical structures of the right hind feet of the mice were observed using an operating microscope. In the CFL + CL transection group, a longitudinal incision was made on the skin over the lateral aspect of the right ankle joint, and the subcutaneous tissue was separated to expose the ankle and subtalar joints. The CFL (Fig. 1A, black arrow on the left) connecting the fibula and the calcaneus was then visible, which was transected with a scalpel. The anterior inferior part of the lateral ankle was explored along the subtalar joint, and the fibular long and short tendons and the extensor digitorum longus tendons were separated using micro tweezers, exposing the CL (Fig. 1A, green arrow on the left) connecting the anterolateral surface of the fibula and the calcaneus; the CL was then transected. In the CL transection group, the CL was exposed and transected in the same manner as described above. In the Sham group, a longitudinal incision was made on the skin over and fascia corresponding to the lateral aspect of the right ankle joint, but without any ligaments being transected. The incision was washed with sterile saline, then the fascia and skin were sutured, and finally the incision was disinfected using balls of cotton wool soaked in Aner iodine. The mice were able to move around and had free access to food and water after awakening in cages. The incisions of the mice were disinfected with cotton wool soaked with Aner iodine twice a day for one week after surgery, and the postoperative condition of mice was closely monitored.

### Balance assessments

The motor coordination and balance of mice were evaluated by measuring their ability to reach a closed safety platform through a beam inclined at 15°. A round wooden beam with a length of one meter and a diameter of 20 mm was used. One end of the beam was connected to the bracket, and the other end was placed on the surface of a workbench and connected to a closed box. A week's training before surgery allowed the mice to skillfully run from one end of the wooden beam to the other, ensuring consistent performance. When each mouse passed through the beam twice in a row within a certain period of time and did not stop on the beam, it was considered to be a valid test. The mice were allowed to pass through the wooden beam for up to 60 s; the duration of two passes through the beam and the number of times the right rear foot slipped out of the wooden beam were recorded as dependent variables [18, 19]. Balance tests were conducted before the operation and three days, one week, four weeks, eight weeks, and twelve weeks after operation. During each test process, each mouse had to complete two tests. Follow-up analysis is based on the average value of the two tests.

### Micro-CT scanning

Twelve weeks after the operation, the mice were killed and the right ankle joint was resected. The skin and part of the soft tissue on the ankle joint surface were removed, and then fixed with 10% neutral formalin. After 48 h of fixation, the specimens were scanned via high-resolution microcomputer tomography (SkyScan 1176, Aartselaar, Belgium) with the following scanning parameters: 50 kV, 500 mA, and 9 um resolution. The scanned image was processed and reconstructed using software. In the software, continuous subchondral bone of the ankle joint and subtalar joint in the region of interest were selected for quantitative analysis of specific indicators such as bone volume fraction and trabecular bone thickness. Subsequently, a three-dimensional reconstructed image was obtained using the software.

### Histomorphometry analysis

After high-resolution microcomputed tomography, all specimens were decalcified in 10% ethylenediamine tetraacetic acid (EDTA) (pH 7.4) solution for one month, and excess soft tissue on the specimens was trimmed and dehydrated in a concentration gradient alcohol. Next, the specimens were transferred to n-butanol for 10 h and soaked in paraffin for 7 h. Finally, according to the standard scheme, a paraffin block wrapped with the specimen was cut into coronal sections (including ankle and subtalar joints) with a thickness of 6 microns using a rotary paraffin slicer. The sections were stained with H&E to evaluate the thickness changes of the ankle and subtalar articular cartilage and subchondral bone, and the modified Mankin score was used to evaluate pathological changes [20]. Changes in the cartilage proteoglycan content were detected by Safranin O-Fast Green staining, and the cartilage of the ankle and subtalar joints were scored histologically using the Osteoarthritis Research Society International (OARSI) OA cartilage histopathology assessment system to quantify the severity of osteoarthritis of ankle and subtalar joint [21]. The content of type II collagen produced by articular chondrocytes was observed via immunohistochemical staining of type II collagen.

### Statistical analysis

SPSS v23.0 (SPSS Inc., Chicago, IL, USA) and GraphPad Prism 8.0 (GraphPad Software, La Jolla, CA, USA) were used for statistical analysis. First, the normality of the distribution and the homogeneity of variance in each group of data were tested. Normally distributed data that had equal variance were analyzed by one-way ANOVA; otherwise, the data were analyzed via Kruskal–Wallis test. The data are expressed as means ± standard deviation (SD). *p* values < 0.05 were considered to be statistically significant [16].

## Results

### Balance assessments

In intergroup comparisons of preoperative balance beam test, no significant difference was observed in the time taken to pass through the balance beam between the three groups (*p* = 0.165).

**Fig. 2:**
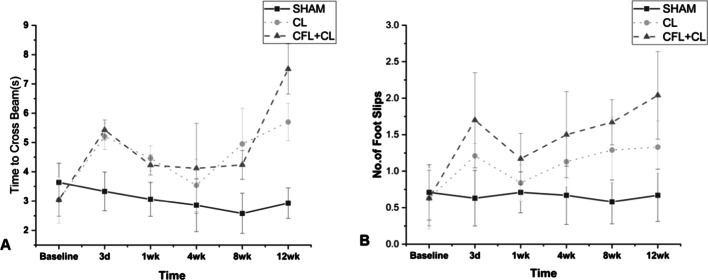
Balance assessments of mice (means ± standard deviations). **A** Time required for mice to cross the balance beam. **B** Number of slips of the right hindfoot when traversing the balance beam

In the postoperative stage, except the fourth week, the time required to pass through the balance beam in the ligament transection groups was significantly longer than that in the Sham group (*p* < 0.05). In comparing ligament transection groups, the time required to pass through the balance beam in the CL transection group was significantly shorter than that in the CL + CFL transection group at 12 weeks after operation (*p* < 0.05) (Fig. 2A). In addition, the transection of ligament also increased the instances of hindfoot slipping in mice. During the preoperative test, there was no statistically significant difference in the number of slips when passing through the balance beam between groups (*p* > 0.05). Compared with the Sham group, the CL + CFL transection group showed a significantly increased number of pulleys passing through the balance beam after operation (*p* < 0.05). There was no statistically significant difference between the CL transection group and Sham group in this regard at one week and four weeks after surgery (*p* > 0.05), but there was statistically significant difference at three days, eight weeks, and twelve weeks after operation (*p* = 0.032, 0.046, and 0.008). Twelve weeks after operation, the mice in the CL + CFL transection group slipped more easily than those in the CL transection group (*p* < 0.05) (Fig. 2B).

### Histomorphometry analysis

**Fig. 3:**
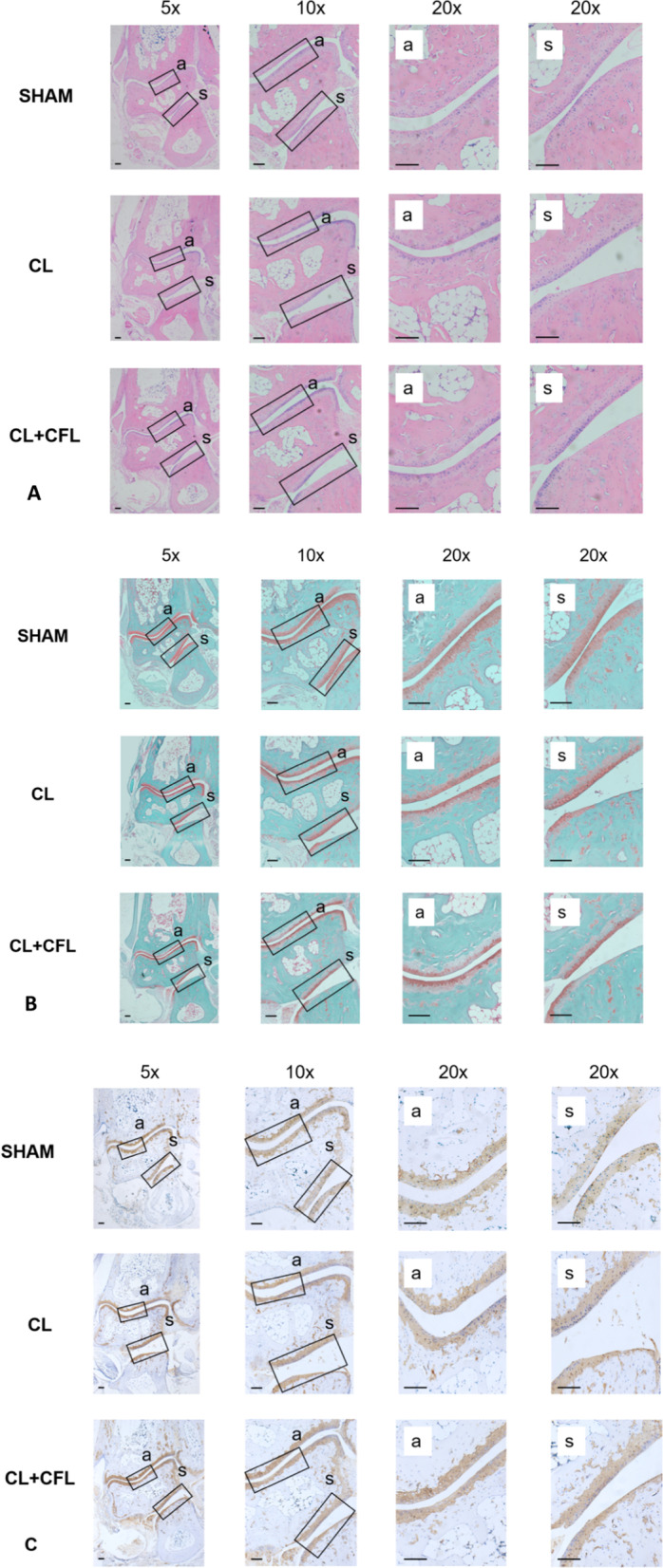
Hematoxylin and eosin (H&E) and Safranin O-Fast green and Type II collagen immunohistochemical staining of the subtalar joints. **A** H&E staining of the subtalar joint in mice. **B** Safranin O-Fast green staining of the subtalar joints in mice. **C** Type II collagen immunohistochemical staining of the subtalar joints in mice. *a* ankle joint, *s* subtalar joint. Scale bar = 100 μm

**Fig. 4:**
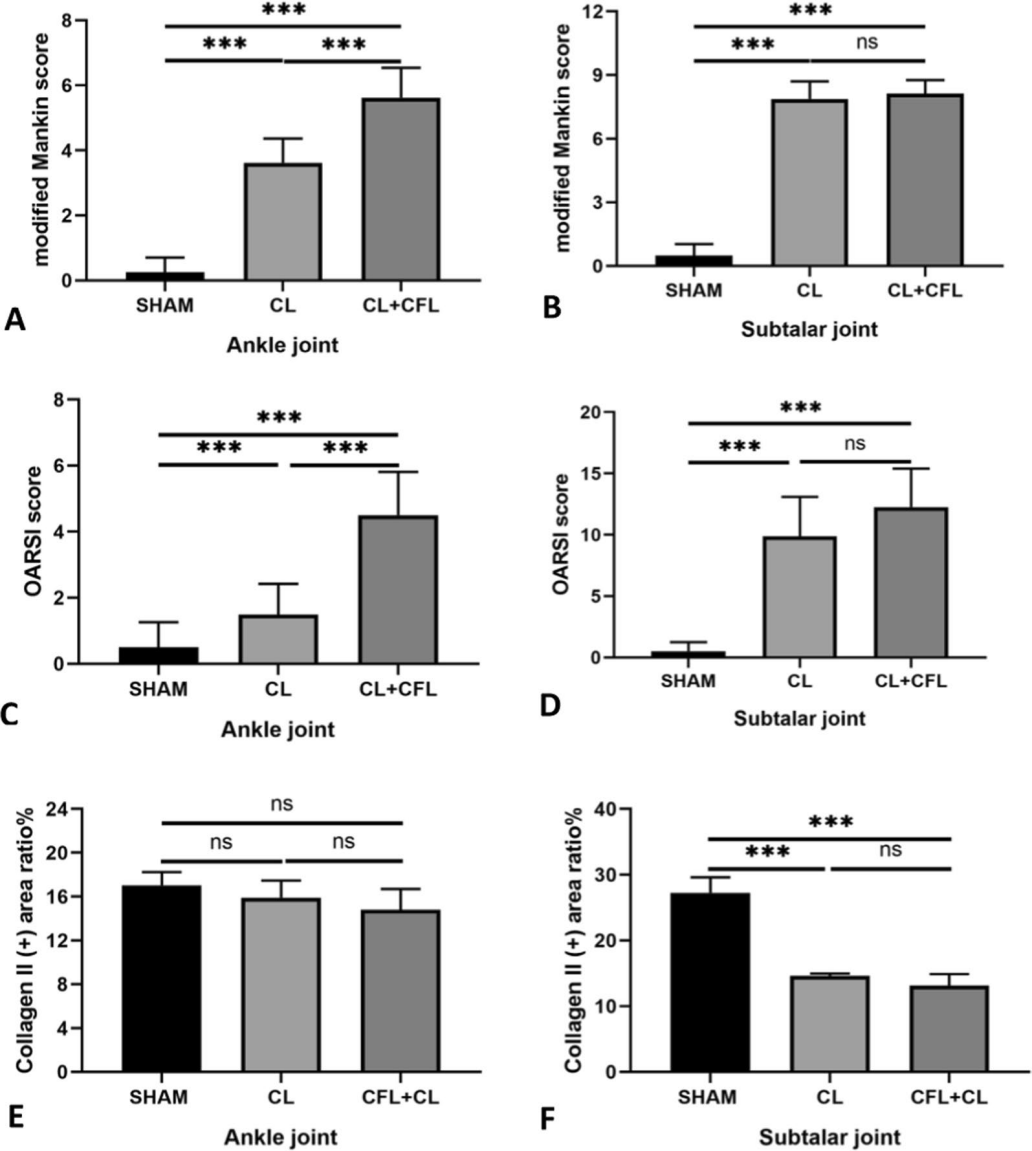
Analysis of the subtalar joints. **A** Modified Mankin scores for the ankle joints in mice. **B** Modified Mankin scores for the subtalar joints in mice. **C** Osteoarthritis Research Society International (OARSI) scores for the ankle joints in mice. **D** OARSI scores for the subtalar joints in mice. **E** Collagen II (+) area ratio percentage for the ankle joints in mice. **F** Collagen II (+) area ratio percentage for the subtalar joints in mice. Statistically significant differences are indicated by *** where *p* < 0.001 between the indicated groups

To further prove that subtalar joint instability can lead to osteoarthritis and destroy the cartilage layer of the subtalar joint and ankle joint, H&E and Safranin O-Fast Green staining were performed. In the typical H&E staining diagram, we can see that the cartilage surface of subtalar joint and ankle joint in Sham group is complete, the morphology of chondrocytes is normal and evenly distributed, and the staining of cell matrix is also normal. In the ligament transection groups, the cartilage layer of subtalar articular surface was obviously missing, stromal staining had reduced, and the cartilage degeneration of ankle joint was relatively light (Fig. 3A). In the typical Safranin O-Fast Green staining diagram, we can see that the cartilage layer of subtalar joint and ankle joint in Sham group is intact, the morphology is complete, the cell matrix structure is normal and the cell morphology is complete. In the ligament transection groups, the defect area of subtalar articular cartilage layer was more than 50%, resulting in local staining failure; the surface of ankle cartilage layer was discontinuous and local cartilage layer was thinner (Fig. 3B). The modified Mankin score and OARSI score in the ligament transection group were significantly higher than those in the Sham group; the difference was statistically significant (*p* < 0.001) (Fig. 4A–D). In the typical immunohistochemical staining diagram of type II collagen, we can see that the content of type II collagen in the cartilage layer of subtalar joint and ankle joint in the Sham group is uniform. However, in the ligament transection group, the content of type II collagen in the subtalar articular cartilage layer was reduced and defective, and the content of type II collagen in the ankle articular cartilage layer was relatively uniform. Using the ImageJ software to calculate the ratio of type II collagen-positive area to the total visual field area, we can observe that the expression of type II collagen in the subtalar articular cartilage in the ligament transection group was significantly lower than that in the Sham group; the difference was statistically significant (*p* < 0.001) (Figs. 3C, 4E, F).

### Micro-CT

**Fig. 5:**
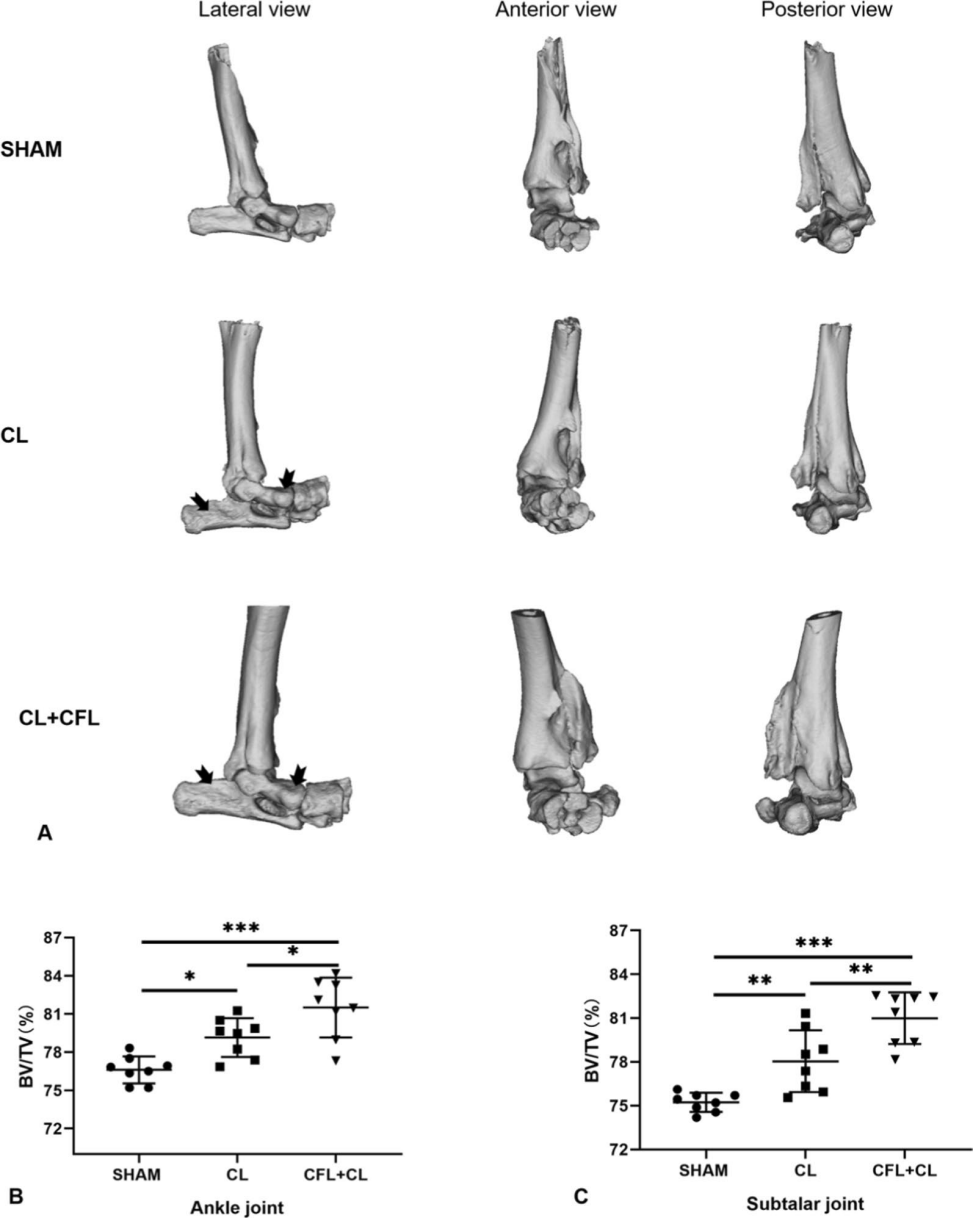
Micro-CT analysis of mouse right feet. **A** Three-dimensional reconstruction of the subtalar joint in mice (lateral view, anterior view, and posterior view) (surface of the joint in the ligament transection group was rough: the part indicated by the black arrow.) **B** Quantitative analysis of bone volume fraction (BV/TV) of the ankle joint in mice. **C** Quantitative analysis of bone volume fraction (BV/TV) of the subtalar joint in mice. Statistically significant differences are indicated by *p < 0.05, **p < 0.01, ***p < 0.001 between the indicated groups

Twelve weeks after the operation, the cartilage layer of the ankle joint surface and subtalar joint surface in the right hind limb of the mice was examined via micro-CT to quantitatively evaluate the subchondral bone. As shown in Fig. 5, the subtalar articular surface in the ligament transection group was rough and severely worn, accompanied by the formation of osteophytes, indicating that the subtalar joint was degenerated. Further, the bone volume fraction (BV/TV) in the CL + CFL transection group and CL transection group were significantly increased compared with those in the Sham group (*p* < 0.05), This showed that the amount of subchondral bone in the subtalar joint and ankle joint had increased, the bone mineral density had increased, indicating a state of "osteosclerosis."

## Discussion

Ankle sprain is one of the most common injuries in sports. A Sprain can lead to the relaxation or rupture of ligaments around the ankle, resulting in a change in the mechanical properties of the ankle or subtalar joint and uneven force distribution around the subtalar joint, and further lead to instability of the ankle or subtalar joint [1–3]. Injuries to the subtalar joint are difficult to detect because of the similarities in the mechanism and clinical manifestations of ankle and subtalar joint injuries [1, 4–7]. At present, researchers are still uncertain about the factors that cause instability of the subtalar joint. Therefore, in this study, the CL alone or CFL combined with CL were transected in mice, trying to establish two animal models of subtalar joint instability. Balance assessments were performed to evaluate whether the motor coordination of mice was damaged, and micro-CT and tissue section staining were performed to evaluate whether long-term subtalar joint instability caused PTOA. The experimental results show that the transection of CFL and CL impairs the motor coordination and balance ability of mice, and such long-term instability can cause traumatic osteoarthritis of the subtalar joint. Thus, this is an effective animal model of subtalar joint instability.

### Ligament protocol

Regarding the ligaments around the subtalar joint, isolated CL injury is not common, because its injury is usually accompanied by the injury of ligaments around the ankle [22]. The CFL is the main stabilizer of the subtalar joint. However, some studies have concluded that tear of the CFL alone increases inversion at the ankle but not at the subtalar joint [9, 10]. In a neutral position, a 140% increase in laxity was found at the ankle after transecting the CFL, while the stability of subtalar joint has not significantly changed [23]. Therefore, our study aims to explore the effect of the transection of CL alone or transection of CL and CFL on subtalar joint instability to provide a new reference for the clinical diagnosis and treatment of subtalar joint instability.

### Balance assessments

The time required to pass through the balance beam for three days and one week in the ligament transection group was significantly longer than that in the Sham group. This may be because there is obvious edema and pain in the foot and ankle of mice in the early post-surgery period, resulting in a decrease in the crawling speed of mice on the balance beam due to pain feedback and an increase in time-consuming accordingly, which suggests that physical activity should be reduced in the early stage after acute ankle injury and as reported in Hubbard-Turner study, early rest after severe ankle sprain is essential to restore the level of physical activity [24]. Four weeks after the operation, there was no statistically significant difference in the time of required for passing through the balance beam between the ligament transection group and the Sham group. At this time, the surgical incision on the mouse foot and ankle had been completely healed and the soft tissue swelling had completely subsided. At the later stage of the study (8 weeks later), the time required for the mice in the ligament transection group to pass the balance beam was significantly longer than that in the Sham group, which was consistent with observations by Hubbard-Turner after transection of the lateral ankle ligament [25, 26]. At this time, there were obvious deformities in the foot and ankle of the mice in the ligament transection group and the joints were stiff, which may reduce their speed while passing through the balance beam. The time required by mice in the CL + CFL transection group for passing through the balance beam was significantly longer than that required by mice in the CL transection group. The reason for this difference may be that the fracture of CFL exacerbated the injury of the subtalar joint.

With regard to the incidence of slipping on the balance beam, the number of slip in the CL + CFL transection group was significantly higher than that in the Sham group, and the number in CL transection group was significantly higher than that in the Sham group at three days, eight weeks, and 12 weeks after surgery. After 12 weeks, the number of slip in the CL group increased by 2.13-fold, and 3.13-fold in the CL + CFL group. In addition, the number of slip in the CL + CFL group 12 weeks after surgery was 1.47-fold that of the CL group, which had same trends with that observed by Hubbard-Turner [25, 26]. The reason for this difference may be that the simultaneous transection of CL and CFL causes more serious injury to the foot and ankle, resulting in more severe tissue edema and a longer duration of edema CFL fracture may aggravate the instability of the subtalar joint and lead to an increase in the number of slips. It also reflects that CFL plays an important role in maintaining the stability of the subtalar joint [9–11].

Twelve weeks after operation, the time required for passing through the balance beam and the instances of slipping from the balance beam in the ligament transection group were significantly higher than those in the Sham group, and the motor balance function was significantly damaged in the ligament transection group. By this time, the mice may have developed long-term chronic subtalar joint instability to post-traumatic subtalar osteoarthritis. Numerous studies have reported that individuals with post-traumatic subtalar osteoarthritis have balancing disorders and movement pattern changes, and there are static and dynamic balance defects in a series of postural controls. These findings are consistent with the results of the mouse model in this study [27–29].

### Histomorphological staining

H&E and Safranin O-Fast green staining showed obvious discontinuities and large defects on the surface of subtalar joint in the ligament transection group. The number of chondrocytes on the joint surface decreased and disappeared, and hollow-nucleated osteocytes were observed, which suggested that the transection of ligament leads to the instability of the subtalar joint, and the instability of subtalar joint without effective treatment can cause PTOA, similar to the previous results of constructing the ankle joint model by cutting the ligament around the ankle joint in mice [15, 16]. In the CL + CFL transection group, except for the defect on the surface of subtalar joint, slight roughness and defect were also seen on the surface of the ankle joint. The possible reason for this phenomenon is that CFL is the only ligament connecting the tibial joint and subtalar joint at the same time. The transection not only causes damage to the subtalar joint but also damages the ankle joint to some extent [9–11, 16]. Immunohistochemical staining of type II collagen indicated that the positive area of type II collagen in the subtalar articular cartilage layer of the ligament transection group significantly decreased. The reason for this difference would be due to degenerated articular chondrocytes, resulting in the failure to normally produce type II collagen, which is consistent with the results of the above two staining methods and similar to the results of Liang et al., who constructed a rat PTOA model through ankle fractures [30].

### Micro-CT

Under normal physiological conditions, the bone resorption effect of osteoclasts and the osteogenesis effect of osteoblasts maintain the dynamic balance of the bone state [31, 32]. Quantitative analysis of micro-CT showed that the bone volume fraction of the subtalar joint in CL + CFL transection group and CL transection group was 5.8% and 2.8% higher than that in the Sham group, respectively; the ankle bone volume fractions in the CL + CFL transection group and CL transection group were 4.9% and 2.5% higher than that in the Sham group, respectively. These results indicate that the partial ligament of the right hind limb of the mouse is broken, which breaks the stable bone balance, resulting in the abnormal activity of osteoblasts, increase in bone density, bone hyperplasia, subchondral bone sclerosis and thickening, and degenerative changes in the joint [15, 33]. This is similar to the CT analysis results of Chang et al. who established an ankle osteoarthritis model by surgery [15]. In addition, during CT image reconstruction and analysis, we found that the ankle joint of mice also had degeneration, which was consistent with previous studies that severe foot instability would cause degeneration of adjacent joints [16, 34], suggesting that the subtalar joint instability of the animal model in this study was serious.

### Limitations

This study has some limitations. First, the animals used in the experiment were mice. Although the structure of the hind foot and ankle of mice is similar to that of humans, strictly speaking, mice are quadruped, whereas humans are bipedal, and the forces borne by the joints during exercise are not completely consistent. Second, the ankle joint and subtalar joint surface of mice are very small, and the cartilage layer area is also thin; accordingly, it is impossible to extract the corresponding cartilage cells and further elaborate on the signaling pathway and molecular mechanism of joint degeneration at a cellular level. Finally, PTOA is a chronic degenerative disease, the study period of only 12 weeks is not long enough for subtalar osteoarthritis development, and an experiment of a longer duration is required to observe the relationship between CSI and subtalar PTOA. It is believed that more reasonable animal models will be developed in future research.

## Conclusions

In this study, we successfully established a mouse model of subtalar PTOA caused by subtalar joint instability by disconnecting the subtalar periarticular ligaments (CFL and CL), which could provide a new understanding of how acute foot ligament injury develops into chronic articular cartilage degeneration and paves the way for the related research on subtalar osteoarthritis in the future.

## Data Availability

The datasets used and/or analyzed during the current study are available from the corresponding author on reasonable request.

## Abbreviations

CL: Cervical ligament
CFL: Calcaneofibular ligament
PTOA: Post-traumatic osteoarthritis
AS: Ankle sprain
CSI: Chronic subtalar instability
CAI: Chronic ankle instability
ITCL: Interosseous talocalcaneal ligament
DL: Deltoid ligament
H&E: Hematoxylin and eosin
SPF: Specific pathogen-free
EDTA: Ethylenediamine tetraacetic acid
OARSI: Osteoarthritis Research Society International
SD: Standard deviation
